# Supplementary material for: MetaQTL: a package of new computational methods for the meta-analysis of QTL mapping experiments
Source: BMC Bioinformatics. 2007 Feb 8;8:49. doi: 10.1186/1471-2105-8-49 (PMC1808479; doi:10.1186/1471-2105-8-49)
Supplement: Additional File 7 — MetaQTL Package : jar file and tutorial. This Zip archive contains both the MetaQTL JAR file and the files of the tutorial. [file 1471-2105-8-49-S7.zip › org.inra.metaqtl/doc/org/metaqtl/factory/package-summary.html]

org.metaqtl.factory


|  |  |  |
| --- | --- | --- |
| **Overview** \| **Package** \| Class \| **Use** \| **Tree** \| **Deprecated** \| **Index** \| **Help** | |  |
| **PREV PACKAGE**   **NEXT PACKAGE** | **FRAMES**    **NO FRAMES**     **All Classes** |  |


---

## Package org.metaqtl.factory

| **Class Summary** | |
| --- | --- |
| **DubiousLocusFactory** | A factory to read a list of dubious markers from a file. |
| **EMResultFactory** |  |
| **MapDBFactory** | A factory to read the list and the properties of a set of genetic maps. |
| **MetaDicoFactory** |  |
| **MetaGraphParFactory** |  |
| **MetaMapSummaryFactory** | A factory to write out the information on a `MetaMap`. |
| **MetaQtlAnalysisFactory** | A factory to deal with the result of a QTL meta-analysis. |
| **MetaQtlAnalysisModelFactory** |  |
| **MetaQtlAnalysisSummaryFactory** | A factory to deal with QTL meta-analysis result. |
| **MetaQtlModelFactory** |  |
| **TreeFactory** | A factory to deal with distance trees. |

---


|  |  |  |
| --- | --- | --- |
| **Overview** \| **Package** \| Class \| **Use** \| **Tree** \| **Deprecated** \| **Index** \| **Help** | |  |
| **PREV PACKAGE**   **NEXT PACKAGE** | **FRAMES**    **NO FRAMES**     **All Classes** |  |


---
